# (2,9-Dimethyl-1,10-phenanthroline-κ^2^ *N*,*N*′)diiodidocadmium

**DOI:** 10.1107/S1600536811044667

**Published:** 2011-11-02

**Authors:** Ismail Warad, Ahmed Boshaala, Saud I. Al-Resayes, Salem S. Al-Deyab, Mohamed Rzaigui

**Affiliations:** a Department of Chemistry, King Saud University, PO Box 2455, Riyadh 11451, Saudi Arabia; b Petrochemical Research Chair, College of Science, King Saud, University, Riyadh, Saudi Arabia; c Laboratoire de Chimie des Matériaux, Faculté des Sciences de Bizerte, 7021 Zarzouna Bizerte, Tunisia

## Abstract

In the title compound, [CdI_2_(C_14_H_12_N_2_)], the mol­ecule sits on a crystallographic twofold axis. The coordination sphere of the Cd^II^ atom is built of two symmetry-equivalent N atoms of one 2,9-dimethyl-1,10-phenanthroline (dmphen) ligand and two symmetry-equivalent I atoms, thus forming a distorted tetra­hedral geometry. Inversion-related mol­ecules inter­act along the *c*-axis direction by π–π stacking inter­actions between the phenanthroline ring systems, with centroid–centroid distances of 3.707 (9) and 3.597 (10) Å.

## Related literature

For coordination chemistry of phenanthroline derivatives and their applications, see: Miller *et al.* (1999 ▶); Bodoki *et al.* (2009 ▶); Kane-Maguire & Wheeler (2001 ▶); Shahabadi *et al.* (2009 ▶). For related structures involving 2,9-dimethyl-1,10-phenanthroline, see: Alizadeh *et al.* (2009 ▶); Preston & Kennard (1969 ▶); Wang & Zhong (2009 ▶). For background information on π–π stacking inter­actions, see: Janiak (2000 ▶).
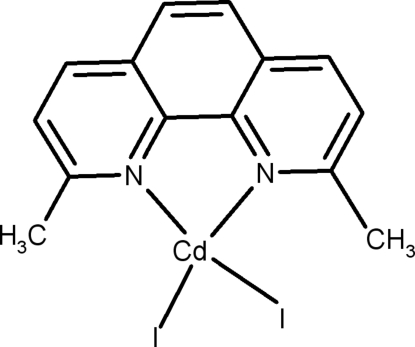


## Experimental

### 

#### Crystal data

[CdI_2_(C_14_H_12_N_2_)]
*M* *_r_* = 574.46
Monoclinic, 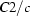

*a* = 15.690 (3) Å
*b* = 11.580 (2) Å
*c* = 9.836 (5) Å
β = 114.65 (4)°
*V* = 1624.3 (9) Å^3^
*Z* = 4
Ag *K*α radiation
λ = 0.56087 Å
μ = 2.72 mm^−1^
*T* = 293 K
0.35 × 0.23 × 0.19 mm

#### Data collection

Enraf–Nonius CAD-4 diffractometer
Absorption correction: multi-scan (*SORTAV*; Blessing, 1995 ▶) *T*_min_ = 0.563, *T*_max_ = 0.605
6126 measured reflections
3986 independent reflections
2306 reflections with *I* > 2σ(*I*)
*R*_int_ = 0.020
2 standard reflections every 120 min  intensity decay: none

#### Refinement

*R*[*F* ^2^ > 2σ(*F* ^2^)] = 0.047
*wR*(*F* ^2^) = 0.127
*S* = 1.02
3986 reflections
88 parameters
H-atom parameters constrained
Δρ_max_ = 1.65 e Å^−3^
Δρ_min_ = −1.30 e Å^−3^

### 

Data collection: *CAD-4 EXPRESS* (Enraf–Nonius, 1994 ▶); cell refinement: *CAD-4 EXPRESS*; data reduction: *XCAD4* (Harms & Wocadlo, 1995 ▶); program(s) used to solve structure: *SIR92* (Altomare *et al.*, 1994 ▶); program(s) used to refine structure: *SHELXL97* (Sheldrick, 2008 ▶); molecular graphics: *ORTEPIII* (Burnett & Johnson, 1996 ▶) and *DIAMOND* (Brandenburg & Putz, 2005 ▶); software used to prepare material for publication: *WinGX* (Farrugia, 1999 ▶).

## Supplementary Material

Crystal structure: contains datablock(s) I, global. DOI: 10.1107/S1600536811044667/pk2353sup1.cif

Structure factors: contains datablock(s) I. DOI: 10.1107/S1600536811044667/pk2353Isup2.hkl

Additional supplementary materials:  crystallographic information; 3D view; checkCIF report

## Figures and Tables

**Fig. 1.**
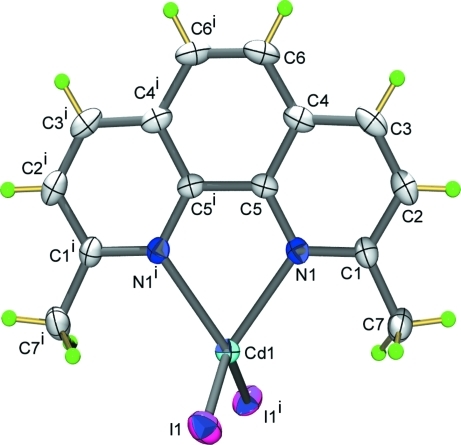
An ORTEP (Burnett & Johnson, 1996) view of (I). Displacement ellipsoids are drawn at the 30% probability level. H atoms are represented as small spheres of arbitrary radii. [Symmetry codes: (i) -x, y, 1/2 - z]

**Fig. 2.**
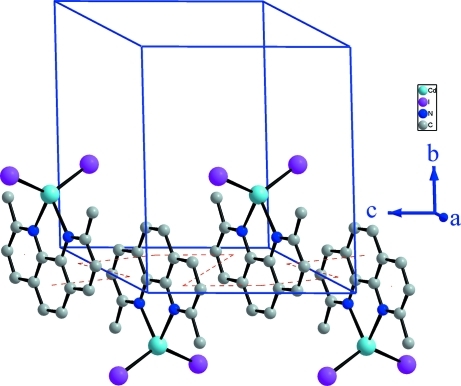
A view of the crystal packing of (I) showing the intermolecular π–π stacking interactions.

## supplementary crystallographic information

### Comment

Metal complexes using 1,10-phenanthroline (phen) and their modified derivative ligands are particularly attractive species for design and developing novel diagnostic and therapeutic agents that can recognize and selectively cleave DNA (Miller *et al.*, 1999; Bodoki *et al.*, 2009). The ligands or the metal in these complexes can be varied in an easily controlled manner to facilitate an individual application, thus providing an easy access for the understanding of details involved in DNA-binding and cleavage (Kane-Maguire & Wheeler, 2001; Shahabadi *et al.*, 2009).  We report herein the synthesis and crystal structure of a new Cd^II^ complex, [CdI2(dmphen)] (I) where dmphen = (2,9-dimethyl-1,10-phenanthroline).

The molecular structure of (I) is shown in Fig. 1. The Cd^II^ cation is located on a special position (1/2, *y*, 1/4) in a tetrahedral environment built up from two nitrogen atoms (N1, N1^i^) of one dmphen bidentate ligand and two iodide ions (I1, I1^i^), [(i): 1 - *x*, *y*, 1/2 - *z*].

Geometrical analysis of the bond lengths and angles around the cadmium atom, Cd–N = 2.305 (3) Å, Cd–I = 2.691 (1)Å and I–Cd–I^i^ = 129.82 (4)°, N–Cd–N^i^ = 73.O5(16)°, N–Cd–I = 112.40 (8)° and N—Cd—I^i^ = 107.48 (8)°, [(i): 1 - *x*, *y*, 1/2 - *z*], shows that the CdI2N2 is distorted. The shortest Cd···Cd distance is 6.650 (2) Å.  Similar coordination geometry around the central atom has been observed in other transition metal complexes such as [HgBr_2_(dmphen)], (Alizadeh *et al.*, 2009), [ZnCl_2_(dmphen)], (Preston & Kennard, 1969), [CuCl_2_(dmphen)] (Wang *et al.*, 2009). The phenyl and pyridyl rings of dmphen ligand are planar with a mean atomic deviation of 0.011 Å and 0.013 Å respectively. The C–C bonds of the two methyl groups are positioned close to the benzene ring plane since the C7–C1–N1–C5 and C7–C1–C2–C3 torsion angles are -179.3 (4)° and -179.5 (5)° respectively.

In the crystal packing the complex molecules are linked together by intermolecular π–π stacking interactions between the pyridyl N1C5C4C3C2C1 (of centroid *Cg*1) and phenyl C5C4C6C6^i^C4^i^C5^i^ [symmetry code: (i) 1 - *x*, *y*, 1/2 - *z*] (of centroid *Cg*2) rings. The centroid–centroid distances between *Cg*1···*Cg*2^ii^ and *Cg*2···*Cg*2^ii^ [symmetry code: (ii) 1 - *x*, -*y*, 1 - *z*] are 3.707 (9) and 3.597 (10)Å respectively, which is less than the 3.8 Å maximum value regarded as relevant for π–π interactions (Janiak, 2000).

### Experimental

A mixture of 2,9-Dimethyl-1,10-phenanthroline (50.0 mg, 0.24 mmol) in dichloromethane (5 ml) and CdI2 (87.9 mg, 0.24 mmol) in methanol (10 ml) was placed in a round bottom flask and stirred for 4 h at room temperature. The solution was concentrated to about 1 ml under reduced pressure. Addition of 40 ml of n-hexane caused the precipitation of white powder, which was filtered and then dried under vacuum to 108 mg (yield 94% based on Cd). The crystal was grown by slow diffusion of diethyl ether into a solution of the complex in dichloromethane.

### Refinement

All H atoms attached to C atoms were fixed geometrically and treated as riding, with C—H = 0.93 Å and 0.96 Å and with *U*_iso_(H) = 1.2Ueq(C) and *U*_iso_(H) = 1.5Ueq(C_methyl_).

### Crystal data

**Table:**

[CdI_2_(C_14_H_12_N_2_)]	*F*(000) = 1056
*M**_r_* = 574.46	*D*_x_ = 2.349 Mg m^−^^3^
Monoclinic, *C*2/*c*	Ag *K*α radiation, λ = 0.56087 Å
Hall symbol: -C 2yc	Cell parameters from 25 reflections
*a* = 15.690 (3) Å	θ = 9–11°
*b* = 11.580 (2) Å	µ = 2.72 mm^−^^1^
*c* = 9.836 (5) Å	*T* = 293 K
β = 114.65 (4)°	Prism, colorless
*V* = 1624.3 (9) Å^3^	0.35 × 0.23 × 0.19 mm
*Z* = 4	

### Data collection

**Table:**

Enraf–Nonius CAD-4 diffractometer	2306 reflections with *I* > 2σ(*I*)
Radiation source: fine-focus sealed tube	*R*_int_ = 0.020
graphite	θ_max_ = 28.0°, θ_min_ = 2.2°
non–profiled ω scans	*h* = −26→25
Absorption correction: multi-scan (*SORTAV*; Blessing, 1995)	*k* = −2→19
*T*_min_ = 0.563, *T*_max_ = 0.605	*l* = −3→16
6126 measured reflections	2 standard reflections every 120 min
3986 independent reflections	intensity decay: none

### Refinement

**Table:**

Refinement on *F*^2^	Primary atom site location: structure-invariant direct methods
Least-squares matrix: full	Secondary atom site location: difference Fourier map
*R*[*F*^2^ > 2σ(*F*^2^)] = 0.047	Hydrogen site location: inferred from neighbouring sites
*wR*(*F*^2^) = 0.127	H-atom parameters constrained
*S* = 1.02	*w* = 1/[σ^2^(*F*_o_^2^) + (0.056*P*)^2^ + 2.6748*P*] where *P* = (*F*_o_^2^ + 2*F*_c_^2^)/3
3986 reflections	(Δ/σ)_max_ = 0.001
88 parameters	Δρ_max_ = 1.65 e Å^−^^3^
0 restraints	Δρ_min_ = −1.30 e Å^−^^3^

### Special details

**Table:**

Geometry. All e.s.d.'s (except the e.s.d. in the dihedral angle between two l.s. planes) are estimated using the full covariance matrix. The cell e.s.d.'s are taken into account individually in the estimation of e.s.d.'s in distances, angles and torsion angles; correlations between e.s.d.'s in cell parameters are only used when they are defined by crystal symmetry. An approximate (isotropic) treatment of cell e.s.d.'s is used for estimating e.s.d.'s involving l.s. planes.
Refinement. Refinement of *F*^2^ against ALL reflections. The weighted *R*-factor *wR* and goodness of fit *S* are based on *F*^2^, conventional *R*-factors *R* are based on *F*, with *F* set to zero for negative *F*^2^. The threshold expression of *F*^2^ > σ(*F*^2^) is used only for calculating *R*-factors(gt) *etc*. and is not relevant to the choice of reflections for refinement. *R*-factors based on *F*^2^ are statistically about twice as large as those based on *F*, and *R*- factors based on ALL data will be even larger.

### Fractional atomic coordinates and isotropic or equivalent isotropic displacement parameters (Å^2^)

**Table:**

	*x*	*y*	*z*	*U*_iso_*/*U*_eq_	
Cd1	0.5000	0.30673 (3)	0.2500	0.04219 (11)	
I1	0.63145 (2)	0.40526 (3)	0.49576 (4)	0.06297 (13)	
N1	0.4331 (2)	0.1468 (3)	0.3059 (3)	0.0385 (6)	
C1	0.3676 (3)	0.1493 (4)	0.3594 (4)	0.0456 (8)	
C2	0.3328 (3)	0.0468 (5)	0.3921 (5)	0.0547 (10)	
H2	0.2873	0.0495	0.4293	0.066*	
C3	0.3655 (3)	−0.0567 (4)	0.3697 (5)	0.0546 (11)	
H3	0.3436	−0.1246	0.3943	0.066*	
C4	0.4325 (3)	−0.0616 (3)	0.3093 (4)	0.0470 (9)	
C5	0.4652 (2)	0.0441 (3)	0.2803 (4)	0.0375 (7)	
C6	0.4684 (4)	−0.1672 (4)	0.2796 (5)	0.0571 (11)	
H6	0.4478	−0.2370	0.3017	0.069*	
C7	0.3344 (3)	0.2640 (5)	0.3834 (6)	0.0615 (12)	
H7A	0.3819	0.2997	0.4698	0.092*	
H7B	0.2782	0.2548	0.3988	0.092*	
H7C	0.3215	0.3116	0.2972	0.092*	

### Atomic displacement parameters (Å^2^)

**Table:**

	*U*^11^	*U*^22^	*U*^33^	*U*^12^	*U*^13^	*U*^23^
Cd1	0.0423 (2)	0.03666 (19)	0.0490 (2)	0.000	0.02046 (17)	0.000
I1	0.0659 (2)	0.0604 (2)	0.0597 (2)	−0.02133 (15)	0.02329 (16)	−0.01232 (14)
N1	0.0341 (13)	0.0426 (16)	0.0372 (15)	−0.0004 (11)	0.0133 (11)	0.0038 (12)
C1	0.0387 (16)	0.057 (2)	0.0416 (19)	−0.0006 (16)	0.0175 (15)	0.0072 (17)
C2	0.0426 (19)	0.073 (3)	0.045 (2)	−0.010 (2)	0.0150 (17)	0.011 (2)
C3	0.054 (2)	0.058 (2)	0.041 (2)	−0.0205 (19)	0.0087 (17)	0.0072 (18)
C4	0.053 (2)	0.0424 (19)	0.0335 (17)	−0.0112 (16)	0.0067 (16)	0.0012 (15)
C5	0.0385 (15)	0.0361 (16)	0.0292 (15)	−0.0038 (13)	0.0053 (12)	0.0004 (13)
C6	0.081 (3)	0.0360 (18)	0.044 (2)	−0.0105 (19)	0.016 (2)	0.0021 (16)
C7	0.059 (2)	0.070 (3)	0.068 (3)	0.015 (2)	0.039 (2)	0.009 (2)

### Geometric parameters (Å, °)

**Table:**

Cd1—N1^i^	2.305 (3)	C3—C4	1.407 (7)
Cd1—N1	2.305 (3)	C3—H3	0.9300
Cd1—I1	2.6907 (14)	C4—C5	1.401 (5)
Cd1—I1^i^	2.6907 (14)	C4—C6	1.427 (6)
N1—C1	1.337 (5)	C5—C5^i^	1.447 (8)
N1—C5	1.355 (5)	C6—C6^i^	1.343 (11)
C1—C2	1.399 (6)	C6—H6	0.9300
C1—C7	1.481 (6)	C7—H7A	0.9600
C2—C3	1.357 (7)	C7—H7B	0.9600
C2—H2	0.9300	C7—H7C	0.9600
			
N1^i^—Cd1—N1	73.05 (16)	C4—C3—H3	119.9
N1^i^—Cd1—I1	107.48 (8)	C5—C4—C3	116.9 (4)
N1—Cd1—I1	112.40 (8)	C5—C4—C6	119.8 (4)
N1^i^—Cd1—I1^i^	112.40 (8)	C3—C4—C6	123.3 (4)
N1—Cd1—I1^i^	107.48 (8)	N1—C5—C4	122.2 (4)
I1—Cd1—I1^i^	129.82 (4)	N1—C5—C5^i^	118.6 (2)
C1—N1—C5	119.9 (3)	C4—C5—C5^i^	119.2 (2)
C1—N1—Cd1	125.3 (3)	C6^i^—C6—C4	121.0 (3)
C5—N1—Cd1	114.9 (2)	C6^i^—C6—H6	119.5
N1—C1—C2	120.6 (4)	C4—C6—H6	119.5
N1—C1—C7	117.5 (4)	C1—C7—H7A	109.5
C2—C1—C7	121.8 (4)	C1—C7—H7B	109.5
C3—C2—C1	120.1 (4)	H7A—C7—H7B	109.5
C3—C2—H2	119.9	C1—C7—H7C	109.5
C1—C2—H2	119.9	H7A—C7—H7C	109.5
C2—C3—C4	120.2 (4)	H7B—C7—H7C	109.5
C2—C3—H3	119.9		
			
N1^i^—Cd1—N1—C1	−179.5 (4)	C2—C3—C4—C5	2.4 (6)
I1—Cd1—N1—C1	78.1 (3)	C2—C3—C4—C6	−178.6 (4)
I1^i^—Cd1—N1—C1	−70.8 (3)	C1—N1—C5—C4	−0.6 (5)
N1^i^—Cd1—N1—C5	0.37 (17)	Cd1—N1—C5—C4	179.5 (3)
I1—Cd1—N1—C5	−102.0 (2)	C1—N1—C5—C5^i^	178.9 (4)
I1^i^—Cd1—N1—C5	109.1 (2)	Cd1—N1—C5—C5^i^	−1.0 (5)
C5—N1—C1—C2	1.3 (6)	C3—C4—C5—N1	−1.2 (5)
Cd1—N1—C1—C2	−178.8 (3)	C6—C4—C5—N1	179.8 (4)
C5—N1—C1—C7	−179.3 (4)	C3—C4—C5—C5^i^	179.3 (4)
Cd1—N1—C1—C7	0.6 (5)	C6—C4—C5—C5^i^	0.3 (6)
N1—C1—C2—C3	0.0 (6)	C5—C4—C6—C6^i^	−1.8 (8)
C7—C1—C2—C3	−179.5 (5)	C3—C4—C6—C6^i^	179.3 (5)
C1—C2—C3—C4	−1.9 (6)		

Symmetry codes: (i) −*x*+1, *y*, −*z*+1/2.
